# Axon guidance molecule semaphorin3A is a novel tumor suppressor in head and neck squamous cell carcinoma

**DOI:** 10.18632/oncotarget.6831

**Published:** 2016-01-08

**Authors:** Zhao Wang, Jie Chen, Wei Zhang, Yang Zheng, Zilu Wang, Laikui Liu, Heming Wu, Jinhai Ye, Wei Zhang, Bing Qi, Yunong Wu, Xiaomeng Song

**Affiliations:** ^1^ Jiangsu Key Laboratory of Oral Diseases, Nanjing Medical University, Nanjing, 210029, China; ^2^ Department of Oral and Maxillofacial Surgery, Affiliated Hospital of Stomatology, Nanjing Medical University, Nanjing, 210029, China; ^3^ Department of Oral Pathology, Affiliated Hospital of Stomatology, Nanjing Medical University, Nanjing, 210029, China

**Keywords:** semaphorin3A, HNSCC, apoptosis, NF-kappaB, Snail

## Abstract

Semaphorin3A (SEMA3A), an axon guidance molecule in the nervous system, plays an inhibitory role in oncogenesis. Here, we investigated the expression pattern and biological roles of SEMA3A in head and neck squamous cell carcinoma (HNSCC) by gain-of-function assays using adenovirus transfection and recombinant human SEMA3A protein. In addition, we explored the therapeutic efficacy of SEMA3A against HNSCC *in vivo*. We found that lower expression of SEMA3A correlated with shorter overall survival and had independent prognostic importance in patients with HNSCC. Both genetic and recombinant SEMA3A protein inhibited cell proliferation and colony formation and induced apoptosis, accompanied by decreased cyclin E, cyclin D, CDK2, CDK4 and CDK6 and increased P21, P27, activated caspase-5 and caspase-7. Moreover, over-expression of SEMA3A suppressed migration, invasion and epithelial-to-mesenchymal transition due in part to the inhibition of NF-κB and SNAI2 in HNSCC cell lines. Furthermore, intratumoral SEMA3A delivery significantly stagnated tumor growth in a xenograft model. Taken together, our results indicate that SEMA3A serves as a tumor suppressor during HNSCC tumorigenesis and a new target for the treatment of HNSCC.

## INTRODUCTION

Head and neck squamous cell carcinoma (HNSCC) is the eighth most common malignancy and accounts for approximately 4.8% of all cancer incidence. An estimated 600,000 new cases of HNSCC are diagnosed worldwide each year, with more than 350,000 deaths annually [1]. HNSCC, which arises from epithelial cells that compose the mucosa of the oral cavity, lips, larynx, pharynx, and nasal passages, is a locally aggressive epithelial neoplasm with a propensity for early metastasis and lymph-node metastasis and a poor prognosis [2, 3]. HNSCC treatment typically involves a multimodal approach comprising systemic chemotherapy, radiation, and surgery. Although advances in these primary treatment modalities have contributed to improvements in the 5-year survival rate (60–65%), many patients still develop recurrent tumors and distant metastases. Once relapse occurs, the 5-year survival rate remains poor (20–40%) [4–6]. Therefore, the identification of new therapeutic targets is important.

The molecular mechanisms underlying malignant progression, such as tumor proliferation, invasion, metastasis and resistance to programmed cell death, are intricate and poorly understood. Epithelial-to-mesenchymal transition (EMT), which has been associated with tumor invasion and metastasis, is frequently observed in HNSCC [7–9]. EMT is a critical early event in tumor progression and is characterized by the down-regulation of epithelial markers (e.g., E-cadherin, β-catenin) and the up-regulation of mesenchymal markers (e.g., Vimentin, N-cadherin) [10]. The EMT process endows epithelial cells with mesenchymal cell properties, reduces intercellular adhesion, and increases the capacity for invasion [11]. The transcription factors Snail and Zeb are representative EMT regulators. They have previously been implicated as inducers of EMT and potent repressors of E-cadherin expression during tumor progression [12–15]. In addition, nuclear factor-kappa B (NF-κB) plays an essential role in both the induction and maintenance of EMT and tumor progression [16–18].

The Semaphorins (SEMAs) were originally described as a large family of conserved axon guidance factors that are crucial for the formation of the nervous system. SEMAs were recently recognized to play a role in various developmental processes, particularly as regulators of cell migration, the immune response, angiogenesis and cancer progression [19]. Class-3 Semaphorins, especially Semaphorin3A (SEMA3A), is involved in the suppression of tumor progression in various types of cancers through binding to its receptor neuropilins (NRPs) [20–24]. SEMA3A also acts as a tumor suppressor because of its distinct antiangiogenic effects [20–22, 25–27]. We previously demonstrated that NRP1 is over-expressed in HNSCC and that its over-expression induces EMT and subsequent migration and invasion through the activation of the NF-κB pathway [28]. However, the expression pattern of SEMA3A and its associated molecular mechanisms in HNSCC have not been explored. In addition, the therapeutic potential of SEMA3A as a new option for HNSCC treatment remains to be clarified.

Therefore, in this study, we detected the expression of SEMA3A in healthy controls and HNSCC patients to investigate its fundamental functions in the tumor progression in HNSCC *in vivo* and *in vitro*.

## RESULTS

### SEMA3A expression is reduced in HNSCC specimens and is associated with poor post-operative overall survival

**Figure 1:**
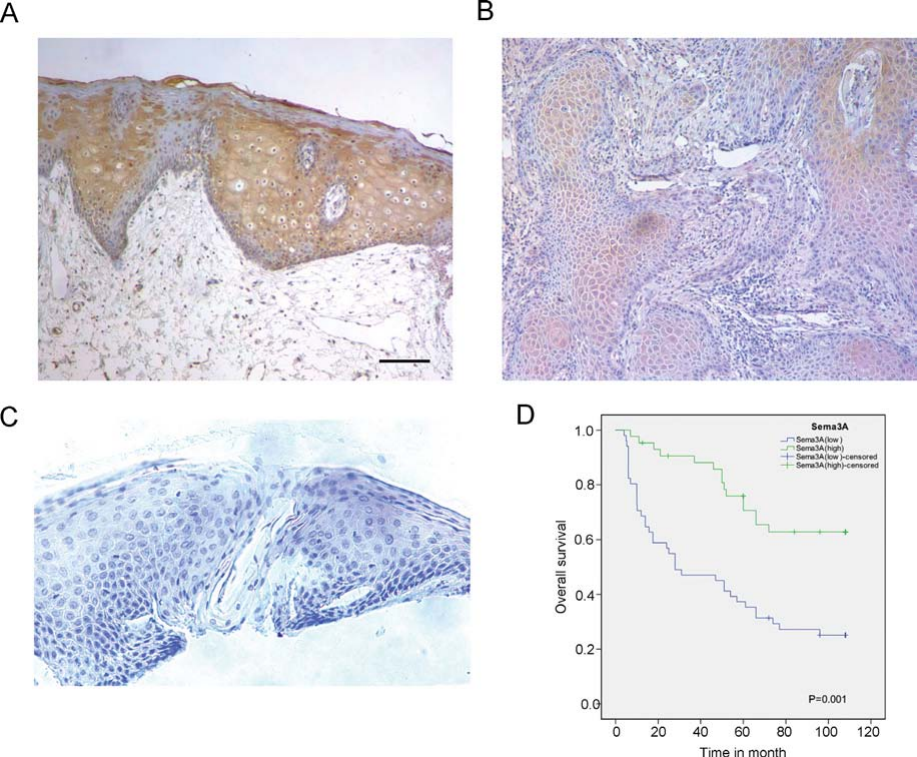
SEMA3A expression is reduced in HNSCC specimens and is associated with a poorer post-operative overall survival (**A**) Immunohistochemistry (IHC) staining for SEMA3A in normal oral epithelium. SEMA3A is highly expressed in normal oral epithelium. (**B**) IHC staining for SEMA3A in HNSCC specimens. SEMA3A is absent or reduced from HNSCC specimens. (**C**) Specimens without incubation with polyclonal antibody served as a negative control. (Scale bar: 100 μm). (**D**) Kaplan-Meier overall survival (OS) curves for 100 patients with HNSCC, according to SEMA3A expression level.

We first examined the expression level of SEMA3A by immunohistochemistry in 100 HNSCC tissue samples and in 20 non-cancerous normal controls. As shown in Table 1, high expression of SEMA3A was observed in 18 of the 20 normal tissues, while among the 100 HNSCC cases, only 43 of the 100 HNSCC specimens exhibited high SEMA3A expression (*P* < 0.001). Furthermore, we evaluated the relationship between SEMA3A expression and the clinical-pathological parameters of the tumor specimens. A significant correlation was observed between SEMA3A expression levels and pathological stage (*P* = 0.002), lymph node metastasis (*P* = 0.017) and tumor *T*-stage (*P* = 0.016). However, no correlation was observed between the levels of SEMA3A staining and age or sex (Table 2). We then analyzed the prognostic data for these patients. Consistent with the data for lymph node metastasis and tumor stage, patients with HNSCC whose tumors had a low level of SEMA3A staining had a poorer prognosis than those whose tumors had a high level of SEMA3A staining (Figure 1). During the follow- up period, among the 100 HNSCC cases, excluding 6 censored samples, 53 patients died of HNSCC or from its complications. According to a univariate analysis, SEMA3A expression, lymph-node metastasis, pathological stage and *T*-stage were associated with overall survival in this patient population (*P* = 0.001, *P* = 0.018, *P* = 0.013 and *P* = 0.034, respectively. Table 3). Multivariate analysis was then performed to determine if the association between SEMA3A and survival was dependent on other factors. The results demonstrated that SEMA3A expression were independently associated with overall survival (*P* = 0.025, Table 3).

**Table 1:** Expression of SEMA3A in normal oral epithelium and HNSCC

	No.	SEMA 3A		*P* value
		High	Low	
HNSCC	100	43	57	< 0.001***
Normal	20	18	2	

Note: 43% of HNSCCs have reduced SEMA3A staining and this is significantly higher than the ratio observed for normal oral epithelium (***P < 0.001, χ^2^-test).

**Table 2:** Correlation of SEMA3A expression and the clinical-pathological parameters of HNSCC specimens

Clinicopatdologic characteristics	No.	SEMA (high)	SEMA (low)	*P* value
**Age**				0.33
**≤ 50**	23	12	11	
**> 50**	77	31	46	
**Sex**				0.25
**Male**	57	28	29	
**Female**	43	15	28	
**Pathological stage**				0.002**
**I–II**	66	44	45	
**III**	34	0	11	
**T-stage**				0.016*
**T1–2**	58	32	26	
**T3–4**	42	13	29	
**N-stage**				0.017*
**N (−)**	57	31	26	
**N (+)**	43	13	30	

Note: The *P* values represent probabilities for SEMA3A expression levels between variable subgroups determined by a χ^2^-test (*P < 0.05, **P < 0.01).

**Table 3:** Univariate and multivariate cox regression analysis of clinical characteristics and SEMA3A expression

Univariate	*P* value	Risk ratio	95% CI	
**Age**	0.285	0.724	0.401	1.308
**Sex**	0.385	0.990	0.966	1.013
**Pathological stage**	0.013*	1.891	1.144	3.125
**T-stage**	0.034*	1.417	1.026	1.958
**N-stage**	0.018*	1.928	1.121	3.315
**SEMA3A expression**	0.001**	0.381	0.209	0.693
**Multivariate**				
**SEMA3A expression**	0.025*	0.428	0.204	0.897

Abbreviations: CI, confidence interval. *P < 0.05, **P < 0.01.

### Endogenous SEMA3A inhibits HNSCC cell proliferation

**Figure 2:**
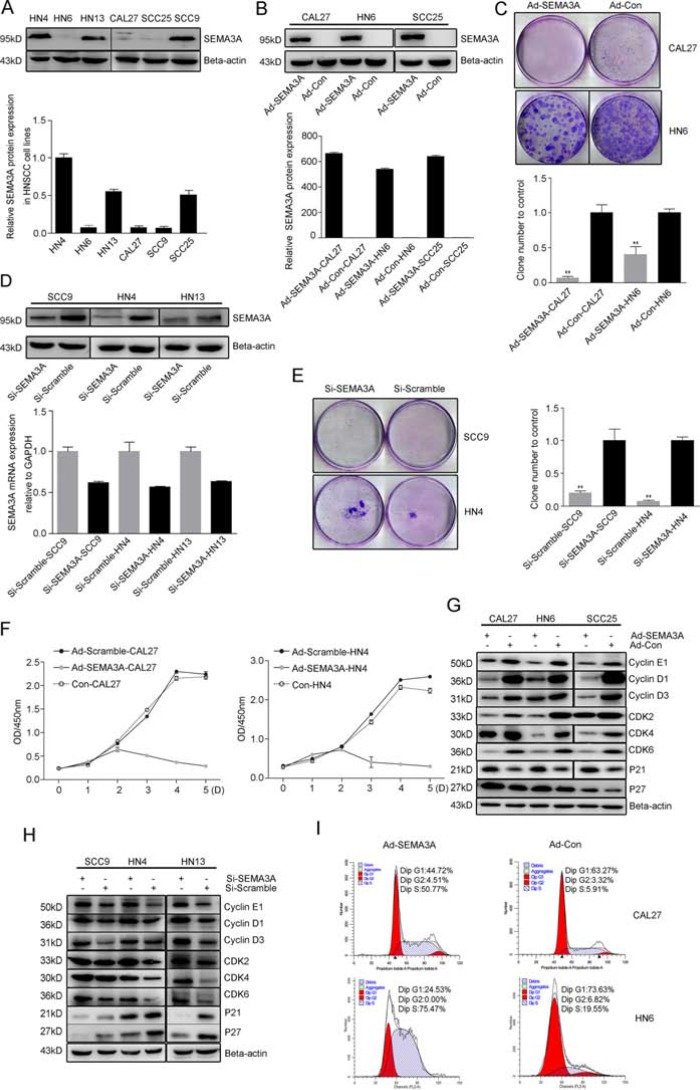
Endogenous SEMA3A inhibits HNSCC cell proliferation (**A**) SEMA3A expression in HNSCC cell lines HN4, HN6, HN13, CAL27, SCC9 and SCC25 assessed by Western blot analysis (up), and quantification of the protein expression (down). (**B**) SEMA3A expression of CAL27, HN6 and SCC25 cells infected with SEMA3A adenovirus on protein (up) and gene (down) levels, detected by Western blot and real-time RT-PCR. (**C**) Images of colonies of SEMA3A-transduced cells (Ad-SEMA3A-CAL27, Ad-SEMA3A-HN6) and control vector (Ad-Con-CAL27, Ad-Con-HN6) stained with crystal violet (up), and the quantification of cell colonies (down). Each data point represents the mean ± SD of data from 3 independent trials. (**P < 0.01) (**D**) Transfection efficiency of SCC9, HN4 and HN13 cells determined by Western blot (up) and real-time RT-PCR (down), after 48 hours’ SEMA3A-siRNA transfection. (**E**) Images of colonies of SEMA3A-siRNA-transfected cells (Si-SEMA3A-SCC9, Si-SEMA3A-HN4) and negative control (Scramble) cells (Si-Scramble-SCC9, Si-Scramble-HN4) stained with crystal violet (left), and the quantification of cell colonies (right). (**F**) Graphs of growth curves of Ad-SEMA3A-cells (grey lines), Ad-Con-cells (black lines) and control cells (dotted lines), as CCK-8 assays carried out over 5 days. (**G, H**) Images of protein expression of CDKs (2, 4, 6), cyclins (E1, D1, D3) and inhibitors of CDKs (P21, P27) in SEMA3A-over-expressed cells (G) or SEMA3A-siRNA-transfected cells (H). (**I**) Flow cytometric analysis of cell-cycle changes in Ad-SEMA3A/Con-CAL27 and Ad-SEMA3A/Con-HN6 cells.

The effect of SEMA3A on HNSCC cells was further investigated in HNSCC cell lines with varying levels of SEMA3A expression. Western blot analysis revealed that the levels of SEMA3A differed across cell lines: HN4, SCC9 and HN13 showed relatively higher SEMA3A expression, while CAL27, HN6 and SCC25 showed lower expression (Figure 2A). We then noted that the expression of endogenous SEMA3A correlated with some phenotypes in the HNSCC cell lines, where CAL27, HN6, SCC25 cells had higher and HN4, HN13, SCC9 cells had lower proliferative, migratory and invasive capacities (Supplementary Figure 1). To establish cell lines with increased expression of SEMA3A, CAL27, SCC25 and HN6 cells were infected with SEMA3A adenovirus. Forty-eight hours after infection, the percentage of infected cells was as high as 80–100% at a MOI of 5 based on GFP fluorescence. In addition, increased SEMA3A expression was detected by Western blot, real-time RT-PCR (Figure 2B) and ELISA assays (Supplementary Figure 2A). Colony-formation assays were performed to determine the effect of SEMA3A on cell proliferation. Compared with cells transfected with control vector (Ad-Con-CAL27, Ad-Con-HN6), SEMA3A-transduced cells (Ad-SEMA3A-CAL27, Ad-SEMA3A-HN6) exhibited a lower colony-formation ability (Figure 2C). Conversely, to establish decreased-SEMA3A expression in cell lines, SCC9, HN4 and HN13 cells were transfected with SEMA3A-specific small interfering RNA (SEMA3A-siRNA); the transfection efficiency was determined by Western blot, real-time RT-PCR (Figure 2D) and ELISA assays (Supplementary Figure 2B). Si-SEMA3A-SCC9 and Si-SEMA3A-HN4 cells exhibited higher colony-formation ability (Figure 2E), suggesting that SEMA3A inhibits HNSCC cell proliferation. To evaluate the toxicity of the adenovirus and to verify the changes in the proliferation of the cells, we determined viability and proliferation of the cell lines using CCK-8 assays. As shown in Figure 2F, compared with control cells (CAL27, HN6), viability and proliferation remained unchanged in Ad-Con-cells (Ad-Con-CAL27, Ad-Con-HN6), whereas significantly lower proliferation ability was observed in Ad-SEMA3A-cells (Ad-SEMA3A-CAL27, Ad-SEMA3A-HN6). In addition, changes in the expression of cell cycle-specific proteins were analyzed by Western blot. As expected, SEMA3A over-expression resulted in the down-regulation of CDKs (2, 4, 6) and cyclins (E1, D1, D3), whereas the expression of P27 and P21 was increased (Figure 2G, Supplementary Figure 3A). Opposite patterns of expression of CDKs, P21 and P27 were observed in SEMA3A-siRNA-transfected cells (Figure 2H, Supplementary Figure 3B). Cell cycle changes were further verified by flow cytometry (Figure 2I), which revealed that Ad-SEMA3A cells were mostly arrested in S-phase of the cell-cycle. These results imply that SEMA3A inhibits HNSCC cell proliferation through impairment of the HNSCC cell cycle.

### SEMA3A over-expression induces apoptosis of HNSCC cells in a caspase-dependent manner

**Figure 3:**
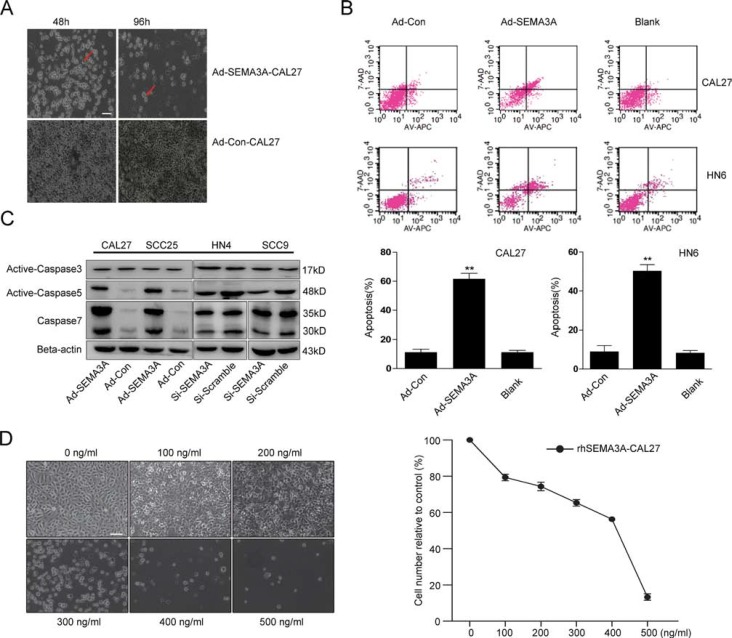
SEMA3A over-expression induces apoptosis in HNSCC cells in a caspase-dependent manner (**A**) A gain of apoptosis-like phenotype of SEMA3A-transfected cells on 48 and 96 hours after transfection. Red arrowheads indicated apoptotic cells. Scale bar: 100 μm. (**B**) Flow cytometric analysis of apoptosis in Ad-SEMA3A/Con-CAL27/HN6. Cells were stained with Annexin V-allophycocyanin (APC) and 7-aminoactinomycin D (7-AAD), followed by FACS (fluorescence-activated cell sorting) analysis (up). Apoptosis was determined by FACS analysis (early apoptotic death cells in lower right plot quadrants and late apoptotic death cells in upper right plot quadrants) and plotted (down). As showed, apoptosis rate in Ad-SEMA3A cells was significantly higher than controls (**P < 0.01, *t*-test). (**C**) Protein changes of caspase (caspase-3, -5, -7) induced by SEMA3A overexpression and depletion were measured by western blotting. Representative images of WB are shown. (**D**) CAL27 cells were treated with rhSEMA3A protein for 48 hours of different concentrations. As the concentration increased, more cells adopted an apoptosis-like phenotype. The remaining cells were counted and plotted. Error bars represent the SD (standard deviation) from three separate experiments. *or**, statistically significant difference compared with the control at *p* < 0.05 or *p* < 0.01, respectively.

Over time, the SEMA3A-transduced cells adopted an apoptosis-like phenotype characterized by cytoplasmic shrinkage and nuclear condensation (Figure 3A). To specifically investigate the role of SEMA3A in cell apoptosis, we performed a flow cytometric analysis 48 hours after infection. As shown in Figure 3B, the rate of apoptosis was significantly higher in Ad-SEMA3A-CAL27 and Ad-SEMA3A-HN6 cells than Ad-Con-CAL27 and Ad-Con-HN6 cells. Caspase (caspase-3, -5, -7) was also detected by Western blot analysis. As anticipated, caspase-5 and caspase-7 were substantially activated, while caspase-3 expression was nearly unchanged (Figure 3C). CAL27 cells also adopted an apoptosis-like phenotype after 48 h of treatment with rhSEMA3A protein as the concentration of rhSEMA3A increased (Figure 3D). These results demonstrate that SEMA3A induced HNSCC cells to undergo apoptosis in a caspase-dependent manner.

### SEMA3A over-expression inhibits tumor growth and induces apoptosis *in vivo*

**Figure 4:**
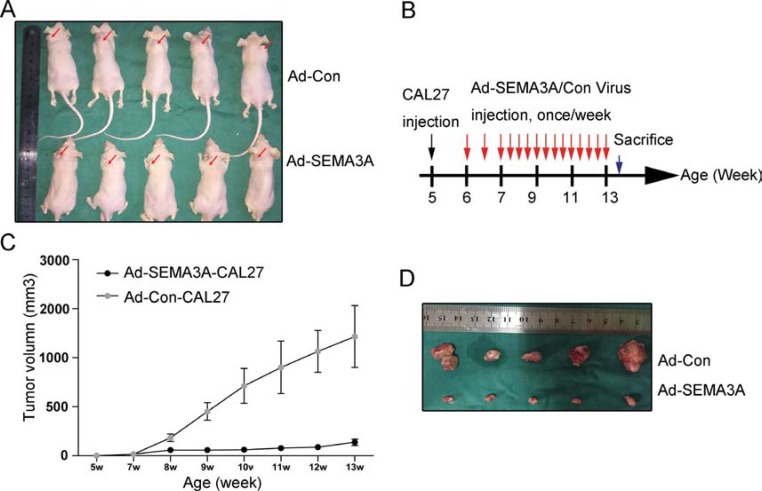
Inhibition of tumor growth and apoptosis *in vivo* induced by over-expression of SEMA3A (**A**) HNSCC xenograft tumor models were established. Images of mice with xenograft tumors were showed 7 weeks after adenovirus injection. Red arrow heads indicated xenograft tumors. (**B**) An illustration of scheme of CAL27 cells injection and adenovirus administration. (**C**) Tumor growth curves of nude mice in Ad-control group (Group 1, *n* = 5) and Ad-SEMA3A group (Group 2, *n* = 5). (**D**) Subcutaneous tumors isolated from nude mice 7 weeks after adenovirus injection.

To further determine if the over-expression of SEMA3A has an effect on tumor growth *in vivo*, an HNSCC xenograft tumor model was successfully established (Figure 4A). Injections of adenovirus were performed as outlined in the general scheme in Figure 4B. The tumor size was recorded every 3 days after injection with CAL27 cells (week 5), as shown in Figure 4C. In the Ad-Con group (Group 1, *n* = 5), all mice developed large tumors with an average size of 1100 mm^3^ by week 13. By contrast, the mice in the Ad-SEMA3A group (Group 2, *n* = 5) developed much smaller tumors with an average size of 108 mm^3^ (Figure 4D). Moreover, we performed TUNEL assay to detect cell apoptosis in xenograft tumor tissues. Apoptosis quantification results revealed that xenograft tumors injected with Ad-SEMA3A adenovirus showed enhanced apoptosis compared with the tumors injected with Ad-Con adenovirus (Supplementary Figure 4). Increased level of SEMA3A and over-expression of cleaved caspase-5 in the Ad-SEMA3A group were also confirmed by immunohistochemistry (data not shown). All the data above suggested that SEMA3A over-expression inhibited tumor growth and induced apoptosis *in vivo*.

### SEMA3A inhibits cancer cell migration, invasion and induces reversion of EMT

**Figure 5:**
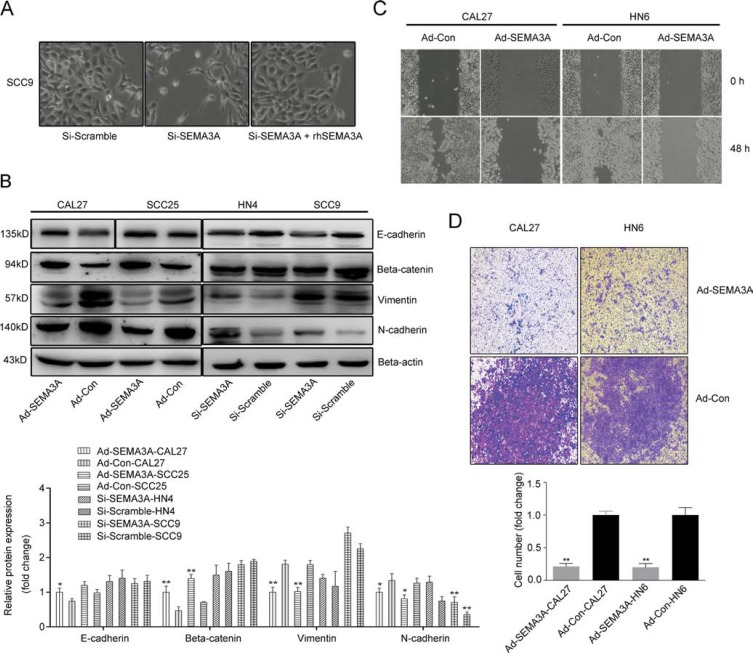
SEMA3A inhibits cancer cell migration and invasion and induces the reversion of EMT (**A**) Cellular morphological changes were shown 48 hours after SEMA3A depletion and rhSEMA3A protein (100 ng/ml) treatment. (**B**) Western blot analysis were used to assess the expression of epithelial (E-cadherin and β-catenin) and mesenchymal (N-cadherin and Vimentin) markers inAd-SEMA3A/Con cells (CAL27, SCC25) and Si-SEMA3A/Scramble cells (HN4, SCC9). (**C**) The differences in the migration ability between the Ad-SEMA3A-CAL27/HN6 and Ad-Con-CAL27/HN6 cells, were measured using the wound-healing assay. (**D**) A transwell assay was employed to analyze the cell invasion ability.

The SEMA3A-depleted cells exhibited a markedly altered cellular morphology characterized by a spindle shape and pseudopodia, suggesting the loss of cell-cell adhesion. By contrast, the cells that were transfected with control siRNA adopted a typical cobblestone-like epithelial morphology; this morphological change was reversed after the SEMA3A-depleted cells were treated with rhSEMA3A protein (100 ng/ml) for 48 h (Figure 5A). These changes demonstrate that depletion of SEMA3A induced an EMT-like phenotype. We then performed Western blot analysis to assess the altered expression of EMT-specific markers at the protein level. As expected, the depletion of SEMA3A induced the down-regulation of the epithelial markers E-cadherin and β-catenin, whereas the expression of the mesenchymal markers N-cadherin and Vimentin was increased (Figure 5B). The opposite pattern of expression was observed in SEMA3A-transfected cells (Figure 5B).

To determine if SEMA3A also inhibits migration and invasion in HNSCC cells, we performed wound-healing and Transwell assays. As shown in Figure 5C, cells transduced with SEMA3A migrated less frequently than cells transduced with the control vector and did not reach confluence after 48 h. Consistent with these results, over-expression of SEMA3A also significantly reduced migratory ability in Transwell assays (Figure 5D). Taken together, these data suggest that SEMA3A inhibits HNSCC cell migration and invasion and induces reversion of EMT.

### SEMA3A-mediated reversion of EMT was associated with inhibition of NF-κB-SNAI2 pathways

**Figure 6:**
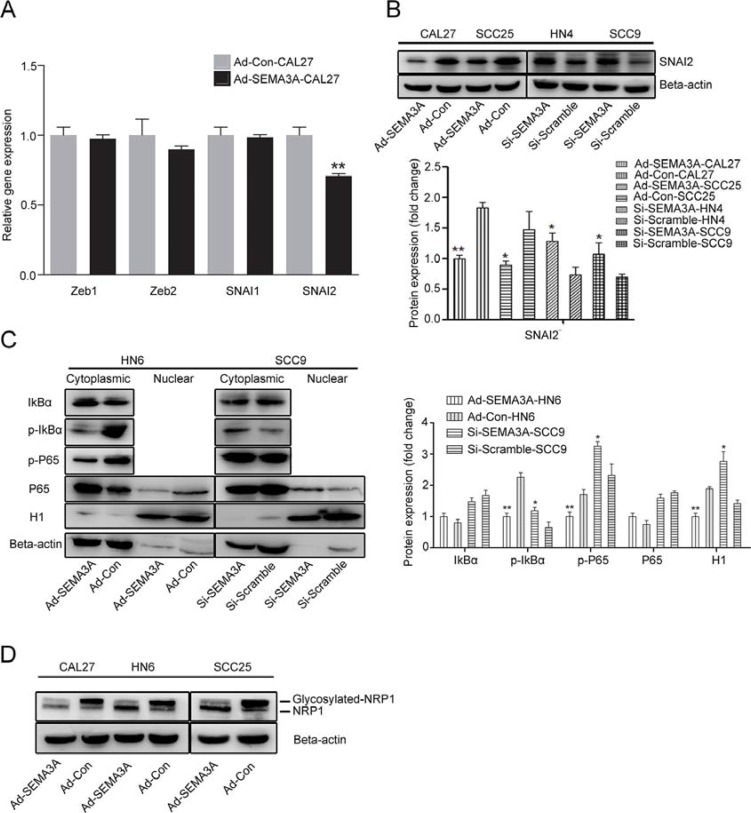
SEMA3A-mediated reversion of EMT was associated with the inhibition of NF-κB-SNAI2 pathways (**A**) Real time RT-PCR was used to detect the expression of classic EMT-inducers Zeb (Zeb1, Zeb2) and Snail (SNAI1, SNAI2) protein in Ad-SEMA3A/Con-CAL27 cells. (**B**) Western blotting was used to detect the expression of SNAI2 protein in Ad-SEMA3A/Con cells (CAL27, SCC25) and Si-SEMA3A/Con cells (HN4, SCC9). (**C**) Western blotting was performed to assess the expression of NF-κB pathway-related proteins in cytoplasmic and nuclear extracts from Ad-SEMA3A/Con-HN6 and Si-SEMA3A/Scramble-SCC9 cells. β-actin and histone H1 were employed as the positive controls for cytoplasmic and nuclear proteins, respectively. Semi-quantitative analysis of changes in protein expression as determined by scanning of the immunoreactive bands. (**D**) A glycosylated form of NRP1 was detected by Western blot analysis.

To further investigate the potential molecular mechanism of EMT, we analyzed the gene expression levels of the classic EMT-inducers Zeb (Zeb1, Zeb2) and Snail (SNAI1, SNAI2) by real-time PCR (Figure 6A). A decrease in SNAI2 gene expression was observed in Ad-SEMA3A cells, while the expression of Zeb1, Zeb2 and SNAI1 was not significantly different in Ad-SEMA3A cells. Decreased SNAI2 expression was observed by Western blot analysis, whereas SEMA3A depletion reverted its effects on SNAI2 (Figure 6B). Components of the NF-κB pathway were also analyzed by Western blot analysis in cells as described previously [28]. As shown in Figure 6C, decreased nuclear translocation of the P65 subunit of NF-κB and reduced phosphorylation of the inhibitor of NF-κB (IκB) were detected in SEMA3A-over-expressing cells; however, SEMA3A-depleted cells exhibited the opposite pattern of nuclear translocation and phosphorylation. Moreover, we detected the components of the NF-κB pathway in xenograft tumor sections by IHC staining. As anticipated, decreased nuclear translocation of P65 and reduced p-IκB were detected in Ad-SEMA3A xenograft tumors, compared with Ad-Con tumors (Supplementary Figure 5). In addition, the expression of the glycosylated form of NRP1 was significantly decreased (Figure 6D, 6E) and Plexins were gradually increased in SEMA3A-over-expressing cells (Supplementary Figure 6). Taken together, these data indicate that SEMA3A-mediated reversion of EMT correlates with the inhibition of NF-κB-SNAI2-dependent pathways in HNSCC cells.

## DISCUSSION

Axon guidance molecules such as the SEMA proteins affect multiple cell types associated with the tumor microenvironment. Recent studies suggest that SEMA3A plays an important suppressive role in the angiogenesis and metastasis of tumors [29]. However, SEMA3A over-expression in several cancer types may promote the dispersal of tumor cells and be associated with metastasis and shorter survival [30, 31]. In the present study, we provide evidence that SEMA3A is abnormally down-regulated in a large fraction of HNSCCs. Its down-regulation is associated with several aggressive characteristics and serves as an independent prognostic factor for patients with HNSCC. Moreover, adenovirus transfection and recombinant SEMA3A protein approaches were used to identify the multiple tumor suppressor roles of SEMA3A and the potential underlying mechanisms *in vitro*. Furthermore, the therapeutic efficacy of SEMA3A-targeted delivery against HNSCC was confirmed in a cancer xenograft model.

In highly invasive and metastatic non-small cell lung cancer (NSCLC), SEMA3A is reduced while the level of MMP-14 is elevated [32]. Similarly, our data revealed a significant decrease in SEMA3A expression in HNSCC compared with normal oral mucosa. Moreover, the expression of SEMA3A was correlated with smaller tumor size and reduced local lymph node metastasis. Chakraborty et al. reported that mouse melanoma cells that over-expressSEMA3A exhibit significant inhibition of cell motility, invasiveness and proliferation. Xenografts derived from SEMA3A-transfected melanoma cells also exhibit poor vascularization and a non-metastatic phenotype [20]. Even more recently, Mishra et al. [33] observed that SEMA3A suppresses tumor growth and angiogenesis through up-regulation of PTEN-dependent FOXO 3a activation. Therefore, our results, together with those of previous studies, indicate that SEMA3A expression is associated with a phenotype of slower proliferation and reduced migration and invasion. The clinicopathological significance of SEMA3A expression in HNSCC prompted us to further dissect the possible biological roles of SEMA3A in cancer progression by gain-of-function assays and genetic approaches.

We over-expressed SEMA3A in HNSCC cell lines that normally express low levels of SEMA3A. MTT assays revealed that the proliferation of these cells was dramatically inhibited. Flow cytometric analysis indicated that the cells were arrested in S-phase of the cell cycle. Accordingly, the expression of the cell cycle-related proteins cyclin A2, cyclin E1, and CDK2/4/6 was reduced in SEMA3A-over-expressing cells. SEMA3A was reported to inhibit the VEGF-mediated up-regulation of cyclin D1 and the induction of cell proliferation in malignant mesothelial cells [34]. Collectively, these results suggest important roles of SEMA3A in the regulation of the cell cycle. In addition to cell cycle dysfunction, increased early-stage apoptosis was also detected by Annexin V-PI double staining after SEMA3A over-expression. The up-regulation of cleaved-caspase-5 and caspase-7 provide further evidence of apoptosis, as do other findings that SEMA3A can sensitize tumor cells to curcumin, an anti-cancer agent that promotes apoptosis and poly ADP ribose polymerase (PARP) cleavage induced by SEMA3A [20]. The over-expression of SEMA3A in our study was induced by adenovirus transfection. To eliminate the possibility that the anti-proliferative effect of SEMA3A was due to the adenovirus, we evaluated the effect of recombinant purified human SEMA3A protein on apoptosis. As expected, as the concentration of SEMA3A increased, the cells exhibited an increase in the severity of apoptosis and cell death. Together with previous findings, our results demonstrate that SEMA3A can induce apoptosis through different mechanisms in cancers, including HNSCC. In conclusion, the anti-proliferative effect of SEMA3A is due to the impairment of the cell cycle and the induction of apoptosis.

In this study, we also observed that the over-expression of SEMA3A impaired the invasion and migration of HNSCC cells. Using multiple experimental tumor models, Casazza et al. [35]. demonstrated that SEMA3A inhibits primary tumor growth and prevents metastatic dissemination via the disruption of angiogenesis and the restriction of tumor cell motility. SEMA3A inhibits cell adhesion and migration through an increase in integrin-α2β1 levels and through the promotion of RhoA translation via a mechanism that involves eIF4E in breast cancer models [36, 37]. Transfection of colon cancer cells with Sema3F, another member of the Sema3 family, reduces integrin-αvβ3 expression and inhibits adhesion to fibronectin [38]. However, these results are primarily based on the molecular mechanisms of SEMA3 proteins in the repulsion and attraction of growth cones of axons in nerve development [39]. By contrast, in HNSCC, EMT plays a vital role in the initiation and progression of metastasis [40, 41]. We therefore hypothesized that SEMA3A can inhibit these processes through the suppression of EMT. Although the epithelial marker E-cadherin was only slightly up-regulated, the mesenchymal markers Vimentin and N-cadherin were significantly down-regulated, indicating the transformation of these cells into a more differentiated state. We previously reported that the over-expression of NRP1 promotes invasion and migration and induces the EMT process through the NF-κB pathway in HNSCC [28]. NRP1 is also the co-receptor of VEGF, and after the formation of its receptor complex VEGFR-NRP, NRP1 promotes cell migration, invasion and EMT through the up-regulation of the NF-κB pathway [42, 43]. SEMA3A can also inhibit EMT and elevated SNAI1 expression induced by sunitinib treatment [26]. Thus, we hypothesized that NF-κB signaling is involved in the inhibition of migration and invasion by SEMA3A in HNSCC. Indeed, we observed that over-expression of SEMA3A down-regulated the NF-κB pathway, whereas knockdown of SEMA3A further up-regulated the NF-κB pathway. Moreover, SNAI2, a downstream nuclear factor of NF-κB, was also down-regulated instead of SNAI1 in SEMA3A-transfected cells. Therefore, for the first time, we confirmed that SEMA3A can suppress invasion and migration through the down-regulation of the NF-κB-SNAI2 pathway in HNSCC.

As mentioned above, we previously demonstrated that NRP1 is elevated in HNSCC and can promote EMT [28]. Because NRP1 is the co-receptor of SEMA3A and is responsible for SEMA3A signal transduction, we examined the pattern of NRP1 expression in SEMA3A-over-expressing cells. Interestingly, the glycosylated form of NRP1 was significantly decreased in SEMA3A-over-expressing cells. Several studies have examined the role of glycosylated NRP1 in cancer. Fukahi et al. [44] first demonstrated the glycosylated pattern of NRP1 and NRP2 in pancreatic ductal adenocarcinoma. Subsequently, Shintani et al. [45] reported similarly modified bands in vascular endothelial cells (ECs) and vascular smooth muscle cells (SMCs), but the patterns were different. They also demonstrated that the glycosylation of NRP1 increased VEGF binding in ECs and SMCs [45]. Accordingly, in our study, the decrease in glycosylated NRP1 may reflect the decrease in NRP1 binding to VEGF because we also observed that VEGFR2, the receptor for VEGF, was down-regulated in SEMA3A-transfected cells (our unpublished data). However, Alattar et al. [46] observed that in esophageal SCC, tumors with positive glycosylated NRP1 were associated with reduced nodal involvement and favorable prognostic stages.

Semaphorins function in both neuronal and non-neuronal cells is mediated by receptor complexes composed of Neuropilin and/or Plexin protein families [47, 48]. It has been well studied that The class 3 Semaphorins do not bind directly to Plexins, but require Neuropilins as obligatory ligand-binding co-receptors for Plexin-based signaling function [49]. In this study, we found that although endogenous expressions of NRP1 and Plexins varied from each other between cell lines, NRP1 seemed to have the same tendency with Plexins in terms of expression level (Supplementary Figure 7A). Furthermore, Plexins (especially A3 and D1) were gradually activated and upregulated after SEMA3A overexpression (Supplementary Figure 7B). Taken together, it might be suggested that SEMA3A perform its functions via NRP1/Plexins-dependent way. Nevertheless, the mechanism by which SEMA3A modulates the expression pattern and activity of NRP1 and, consequently, the phenotype of the tumor requires further study.

In summary, we have demonstrated for the first time that SEMA3A expression is down-regulated to low levels in most human HNSCC and that the over-expression of SEMA3A suppresses the proliferation, invasion and induction of apoptosis of cancer cells both *in vitro* and *in vivo*. Therefore, SEMA3A may function as a tumor suppressor and is a candidate for HNSCC therapy.

## MATERIALS AND METHODS

### Tumor samples and immunohistochemistry

Tumor samples were obtained from 100 patients with HNSCC who were histopathologically and clinically diagnosed at the Stomatological Hospital of Jiangsu Province, Nanjing Medical University, from 2004 to 2011. None of the patients had been treated with any tumor-specific therapies before surgery. The follow-up period ranged from 4 to 108 weeks after surgical resection. Patients who died of other causes were excluded from the analysis. Tissue samples from normal oral lesions were also collected and served as normal controls. Among the 20 normal tissue samples, 13 were obtained from the border of the defect after removal of benign oral neoplasms, and 7 were obtained from freshly injured oral mucosa after trauma. All tissues were obtained with informed consent of the patients. This study was approved by the institutional ethics committee of Nanjing Medical University.

All collected samples were embedded in paraffin, and immunohistochemical analysis of SEMA3A expression was performed as described previously [50]. Briefly, tumor tissue samples were cut into 4-μm-thick sections and placed onto polylysine-coated slides. After deparaffinization and rehydration in water, the slides were treated with 3% hydrogen peroxide to block endogenous peroxidase activity. Then, antigen retrieval was performed in 0.01 M sodium citrate buffer (pH 6.0), followed by incubation with 10% normal goat serum to block non-specific binding sites. The slides were then incubated with the rabbit polyclonal anti-Semaphorin3A antibody (1:100; Abcam, UK) with Two-Step Histostaining Reagent (ZhongshanGoldenbridge Bio). The slides were stained with a DAB substrate (ZhongshanGoldenbridge Bio) and counterstained with Mayer's hematoxylin. Microscopic analysis was performed under a light microscope (Zeiss, Germany) by 2 independent pathologists. Immunoreactivity was semi-quantitatively evaluated on the basis of staining intensity and distribution scores which was calculated as intensity score multiplied by proportion score as we reported previously [28, 51]. The intensity score was measured on a scale of 0–3 (0, negative; 1, weak; 2, moderate; or 3, strong), and the proportion score was measured on a scale of 0–4 (0, < 5% positive cells; 1, 5–20% positive cells; 2, 21–50% positive cells; 3, 51%–80% positive cells and 4, 81–100% positive cells). Individuals were classified into the high-expression group when the SEMA3A expression score was greater than 4; if the score was ≤ 4, the individual was classified into the low-expression group. The scoring of the slides was finished in a double-blind fashion.

### Cell culture and adenovirus infection experiments

Human HNSCC cell lines CAL27, SCC9, and SCC25 were purchased from American Type Culture Collection (ATCC), and human HNSCC cell lines HN4, HN6, and HN13 were obtained from University of Maryland School of Dentistry [52]. All cells were cultured in Dulbecco's Modified Eagle's Medium and Ham's F12 medium supplemented with 10% fetal bovine serum (FBS) and 100 unit/ml penicillin/streptomycin (Invitrogen, Carlsbad, CA, USA) in humidified incubators at 37°C in an atmosphere of 5% CO_2_. For SEMA3A protein treatment, cells at 70–80% confluence were treated with varies concentrations of recombinant human Semaphorin3A protein (PEPROTECH, USA) for 48 h.

The SEMA3A over-expression vector pCMV-SEMA3A-MCS-EGFP encoding full-length human SEMA3A cDNA and the negative control vector pCMV-MCS-EGFP were subcloned into pAdG135 to generate pAdCMV-SEMA3A-MCS-EGFP and pAdCMV-MCS-EFGP. All recombinant vector and adenovirus packaging procedures were performed by GeneChem Co., Ltd (Shanghai, China).

For SEMA3A infection, the CAL27, SCC25, HN6 cell lines were infected with Ad-SEMA3A/Ad-Control adenovirus in the presence of 10 μg/ml polybrene (Sigma, USA) at an appropriate multiplicity of infection (MOI) according to the manufacturer's instructions. 48 hours after infection, the infection efficiency was determined by measuring GFP fluorescence using an inverted fluorescence microscope (DMI3000B, Leica, Germany). The cells were harvested, and the expression level of SEMA3A was determined by real time RT-PCR and Western blot analysis (see below).

### Western blot analysis

Total protein was extracted with lysis buffer (Beyotime, Shanghai, China) containing protease inhibitor cocktail. Cytoplasmic/nuclear proteins were extracted with nuclear and cytoplasmic extraction reagents (KeygenBiotech, Nanjing, China). Proteins were quantified with Coomassie Brilliant Blue, and BSA served as the standard. Proteins were separated by SDS-PAGE on a 10% gel and transferred onto PVDF membranes (Millipore, MA, USA). The membranes were blocked with 5% skim milk at room temperature for 2 h and then incubated with primary antibodies for SEMA3A, NRP1, active-caspase-3, Plexin A1, A2, A3, A4, D1 (Abcam, USA), P65, p-P65, IκB, p-IκB, CDK2/4/6, cyclin E1, cyclin D1/D3, P21, P27, caspase-7 (CST, USA), E-cadherin, N-cadherin, Vimentin, β-catenin, β-actin, H1, cleaved-caspase-5, and SNAI2 (Bioworld, China). All antibodies were used at a 1:1000 dilution. The membranes were incubated with anti-goat IgG HRP-conjugated secondary antibodies (ZhongshanGoldenbridge Bio) for 1 h at room temperature. Immunoreactive bands were detected with Immobilon Western Chemiluminescent HRP Substrate (Millipore) and visualized using an ImageQuantLAS 4000 mini imaging system (General Electric). Analyses of the bands were performed using ImageJ software.

### RNA interference

SCC25, HN4, HN13 cells were cultured at a concentration of 2 × 10^5^ cells per well in six-well plates. Cells at 30–40% confluence were transfected with 10 μM SEMA3A-siRNA or Control-siRNA (Santa Cruz Biotechnology, Inc.) according to the manufacturer's instructions. The transfected cells were incubated for 48 h and then harvested for further analysis.

### Real-time RT-PCR

Total RNA was isolated from cells using TRIzol reagent (Invitrogen), and the resulting mRNA was converted to cDNA using 5 × PrimeScript RT Master Mix (TaKaRa) at 37°C for 15 min and 85°C for 5 s according to the manufacturer's instructions. Quantitative PCR (qPCR) was performed using 5 × SYBR Master Mix (Roche) for 40 cycles in a 7300 ABI Real-Time PCR System (Applied Biosystems, USA) under the following conditions: 95°C for 30 s, 95°C for 5 s, and 60°C for 31 s. Relative mRNA levels were analyzed by the 2^(−ΔΔCt)^ method with GAPDH as the reference gene. All primers were designed and synthesized to target the specific sequences of the genes as follows:

SEMA3A: F:5′-GAGAGTGACAATCCTGAAGATGACAA-3′

R: 5′-TCTGACCTATTCTAGCGTGAGTAGC-3′

Zeb1: F:5′-TACAGAACCCAACTTGAACGTCACA-3′

R:5′-GATTACACCCAGACTGCGTCACA-3′

Zeb2: F:5′-TGCTCGCACTACAATGCATCAG-3′

F:5′-CCTTCACGTCCAGGTCACTTTAAGA-3′

SNAI1: F:5′-TCGGAAGCCTAACTACAGCGA-3′

R:5′-AGATGAGCATTGGCAGCGAG-3′

SNAI2: F:5′-TGTGACAAGGAATATGTGAGCC-3′

R:5′-TGAGCCCTCAGATTTGACCTG-3′

Plexin A1: F:5′-CTCCCTGCCGTGGCTGCTCAACAA-3′

R:5′-ACCACAGTGCGGCCCCGATAGTCA-3′

Plexin A2: F:5′-CTGAGAATCGTGACTGGACCT-3′

R:5′-GCTTATAGACCCGGTTGATGG-3′

Plexin A3: F:5′-AGTCCTGCTATCGTGGGGAG-3′

R:5′-CAGAAGTTGCCGTTGATCTGC-3′

Plexin A4: F:5′-GTCATTTGTCACATTCCGAGGA-3′

R:5′-GCTTGTAAATCCGATTGACGGC-3′

Plexin D1: F:5′-GTCATTTGTCACATTCCGAGGA-3′

R:5′-GCTTGTAAATCCGATTGACGGC-3′

GAPDH: F:5′-GAAGGTGAAGGTCGGAGTC-3′

R:5′-GAGATGGTGATGGGATTTC-3′

### Colony-formation assays

CAL27, HN4, HN6 cells were infected with adenovirus and plated at a density of 1000 cells per well in 35-mm culture plates containing 3 ml of complete medium, followed by incubation at 37°C and 5% CO_2_ for 10 days. Cell colonies were stained with 0.005% crystal violet and analyzed by microscopy.

### Cell migration and invasion assays

*In vitro* cell migration assays were performed in Transwell chambers (8-μm pore size; Costar) according to the manufacturer's instructions. A total of 3 × 10^4^ cells were seeded into the top chamber of each insert and incubated at 37°C for 24 h. Similar inserts coated with Matrigel (BD Biosciences) were used to determine invasive potential in cell invasion assays.

### Cell counting kit-8 (CCK-8) experiments

Ad-SEMA3A/Ad-Control-CAL27 and Ad-SEMA3A/Ad-Control-SCC25 cells were seeded in 96-well microplates at a density of 2 × 10^3^ cells per well. The cells were incubated in new medium containing 10% CCK-8 reaction solution. After incubation for 1 h, the absorbance was measured on a spectrophotometer microplate reader (Multiskan MK3, Thermo, USA) at a wave length of 450 nm, according to the manufacturer's instructions. Five independent experiments were performed.

### Flow cytometry

To analyze apoptosis, cells were harvested 48 h after infection. Flow cytometric analysis of apoptotic cells was performed by staining the cells with Annexin V-allophycocyanin (APC) and 7-aminoactinomycin D (7-AAD) using the Annexin V Apoptosis Detection Kit (BD Pharmingen) for 15 min according to the manufacturer's protocol. For cell-cycle analysis, infected cells were washed in phosphate-buffered saline (PBS) and fixed in 70% ice-cold ethanol for 10 min at 4°C. The cells were then washed twice in PBS, stained with propidium iodide (50 μg/ml) in the presence of 50 μg/ml RNase A (Sigma-Aldrich), and incubated for 1 h at room temperature. The detection of the proportion of apoptotic cells and cell-cycle analysis were performed using a FACSCalibur flow cytometer (BD Biosciences) and CellQuest Pro software (BD Biosciences). The rate of apoptosis and the cell cycle stage in the Ad-SEMA3A cells were compared with those in the Ad-Control cells, which served as the negative control.

### SEMA3A ELISA assays

HNSCC cells were seeded in 24-wells (15,000 cells/well) overnight and were infected with Ad-SEMA3A/Ad-Control adenovirus or transfected with Si-SEMA3A/Si-Scramble RNA as described above. 48 hours later, culture medium were collected to measure the amount of SEMA3A using the human SEMA3A ELISA kit (Elabscience Biotechnology), according to the manufacturer's instructions.

### HNSCC xenograft tumor model and adenovirus injection

Five-week-old male BALB/c-nu mice were purchased from the Nanjing Medical University Animal Research Center. The mice were anesthetized and injected subcutaneously on the dorsa with 2 × 10^6^ CAL27 cells (0.2 ml) suspended in Matrigel (BD). When the tumors reached 3.0–5.0 mm in diameter (6-week-old), the 10 mice were randomly assigned to two groups. In each group, tumors were injected with SEMA3A-adenovirus (10^8^ PFU, group 2, *n* = 5) or Control-adenovirus (10^8^ PFU, group 1, *n* = 5). The injections were performed at several points within the tumor twice a week for 7 weeks. The tumor size was measured with a caliper every 3 days, and the tumor volume was calculated using the following formula: Volume = (L × W^2^)/2, where L equals length and W equals width. Seven weeks after the completion of the series of injections, all mice were sacrificed. The freshly removed samples were fixed in 5% paraformaldehyde solution for 12–24 h and paraffin-embedded for further analysis.

All animal procedures were performed in accordance with the institutional animal welfare guidelines of Nanjing Medical University.

### TUNEL assays

For apoptosis quantification, sections were processed for *in situ* immunolocalization of nuclei exhibiting DNA fragmentation, by the terminal deoxynucleotidyl transferase (TdT)-mediated dUTP nick-end labeling (TUNEL) technique, using the *In Situ* Cell Death Detection Kit, Fluorescein (Roche Applied Science, Switzerland). Sections were treated according to the manufacturer's instructions and observed using an inverted fluorescence microscope (DMI3000B, Leica, Germany). A total of 300 epithelial cells were counted blindly by two independent observers from representative fields and the percentage of TUNEL positive cells was recorded. All percentages were used to get the mean value per group.

### Statistical analysis

Statistical analysis was performed using the SPSS statistical package (version 19.0). Results of quantitative data were expressed as the mean ± SD and evaluated using the Student's *t*-test. For immunohistochemical analysis, Pearson's chi-square (χ^2^) test was used to analyze the distinguishing expression of SEMA3A in normal oral epithelium and HNSCC specimens. The χ^2^ tests were also used to analyze the association between SEMA3A expression and categorical clinical variables. The effect of SEMA3A expression on overall survival was performed using Kaplan–Meier method and compared using log-rank test. Overall survival (OS) was defined as time from surgery to any event of interest. A Cox proportional hazards model was used to identify independent predictors of survival. The rate of apoptosis (early apoptosis and late apoptosis) in the Ad-SEMA3A cells were compared with that in the Ad-Control cells by Student's *t*-test. TUNEL positive cells counting distinction in xenograft groups were also analyzed by Student's *t*-test. All experiments were performed at least three times. All analysis were two-sided, and *P* < 0.05 was considered statistically significant (*P < 0.05, **P < 0.01, ***P < 0.001).

## SUPPLEMENTARY FIGURES AND TABLES


