# Supplementary material for: Axon guidance molecule semaphorin3A is a novel tumor suppressor in head and neck squamous cell carcinoma
Source: Oncotarget. 2016 Jan 8;7(5):6048–62. doi: 10.18632/oncotarget.6831 (PMC4868739; doi:10.18632/oncotarget.6831)
Supplement: Supplementary file 1 [file oncotarget-07-6048-s001.pdf]

Axon guidance molecule semaphorin3A is a novel tumor suppressor in head and neck squamous cell carcinoma

Supplementary Materials

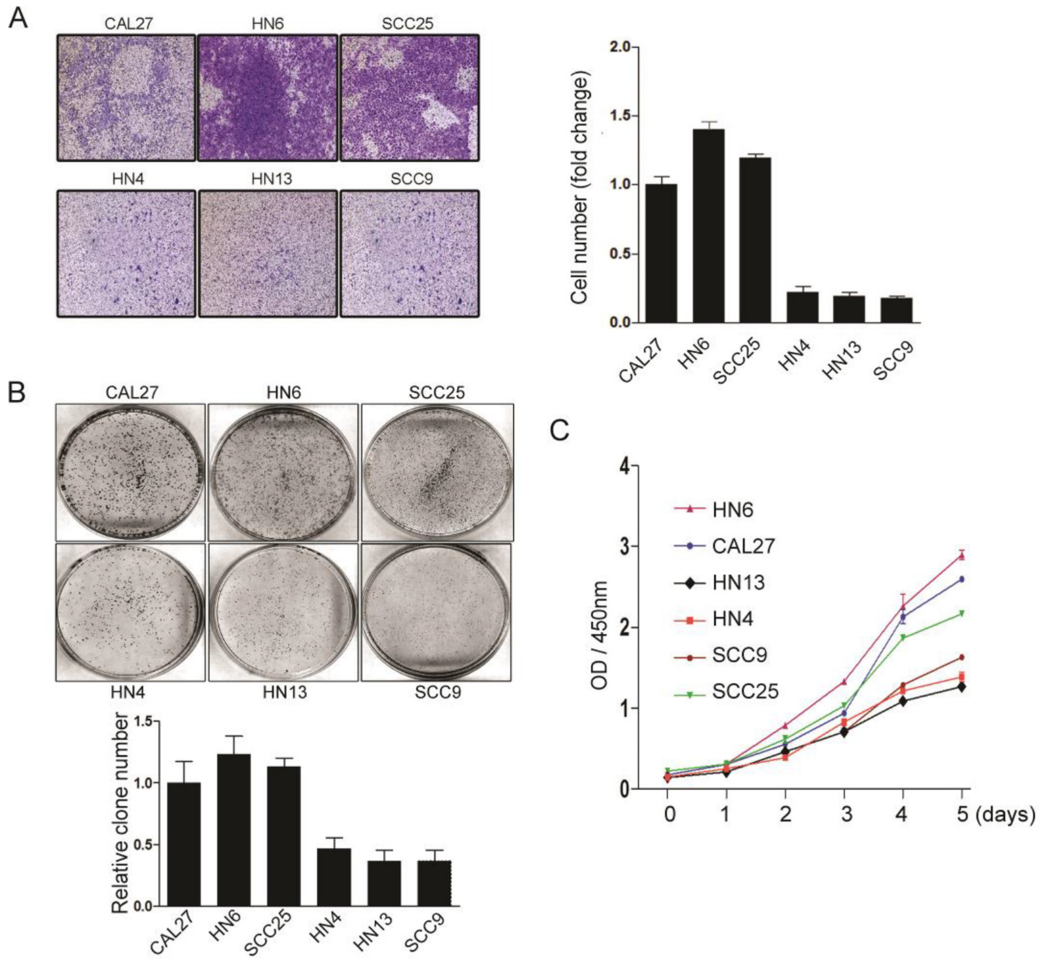

Supplementary Figure S1: The expression of endogenous SEMA3A correlates with phenotypes of the human HNSCC cell lines. (**A**) Transwell invasion assays for 6 HNSCC cell lines (left). Quantification analysis (right) showed that CAL27, HN6 and SCC25 cells had higher invasive ability compared with HN4, HN13 and SCC9 cells. (**B**) Colony-formation assays for 6 HNSCC cell lines (up). Quantification analysis (down) demonstrated that CAL27, HN6 and SCC25 cells had higher proliferative ability compared with HN4, HN13 and SCC9 cells. (**C**) Growth curves of 6 HNSCC cell lines plotted from CCK-8 assays. The proliferative ability of CAL27, HN6 and SCC25 cells was higher than HN4, HN13 and SCC9 cells.

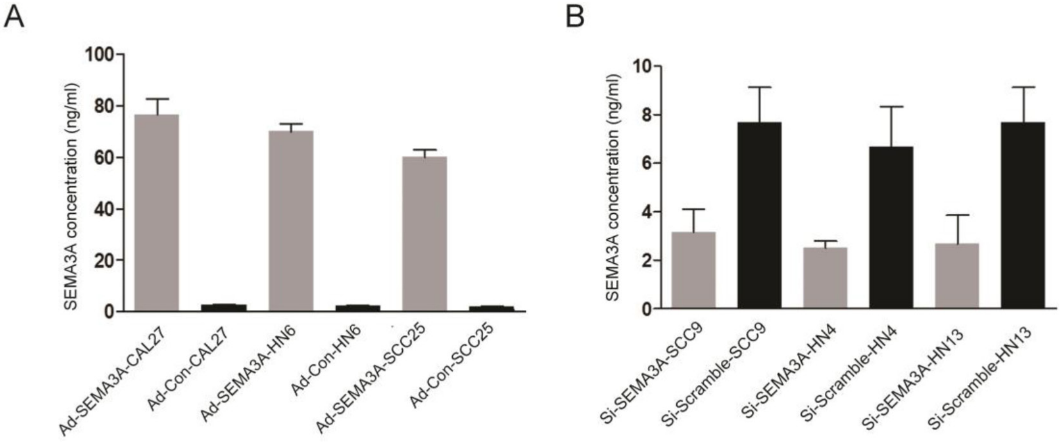

Supplementary Figure S2: Endogenous SEMA3A concentration in conditioned media measured by ELISA assays in the human HNSCC cell lines. (**A**) SEMA3A concentrations in culture medium for Ad-SEMA3A/Con cells were determined using Human SEMA3A ELISA Kit. SEMA3A concentration in conditioned media was much higher after cells were infected with SEMA3A adenovirus. (**B**) SEMA3A concentration in conditioned media was decreased after SEMA3A was depleted by Si-RNA.

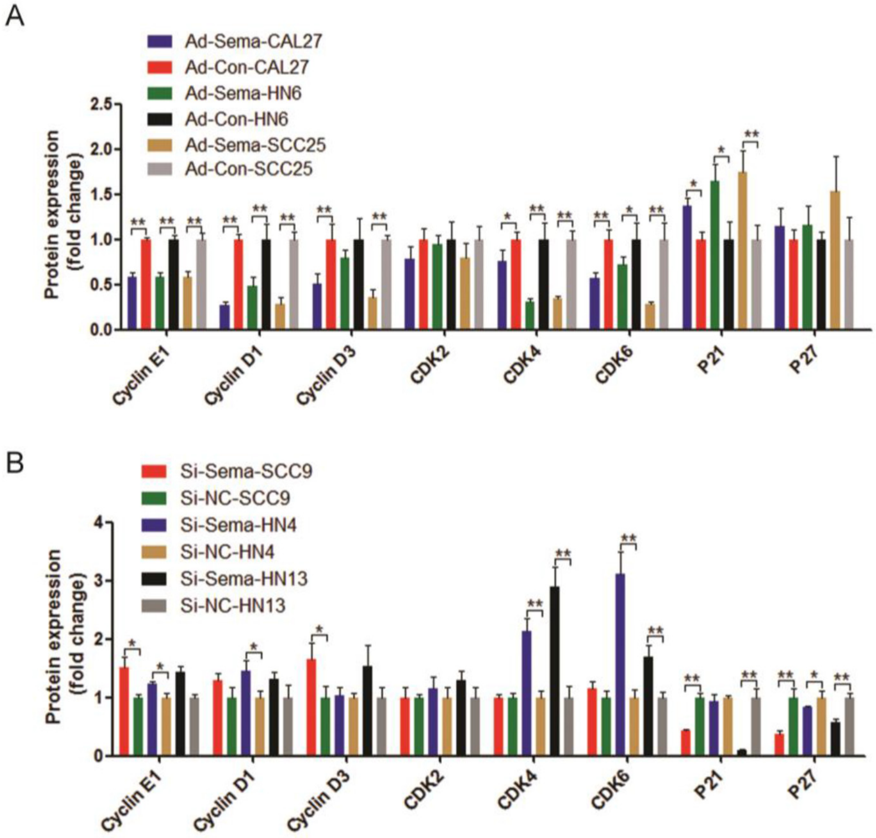

Supplementary Figure S3: Semi-quantitative analysis of changes in cell-cycle related protein expression. (**A**) CDKs (2, 4, 6) and cyclins (E1, D1, D3) were down-regulated, whereas the inhibitors P27 and P21 were increased after CAL27 and HN6 cells were infected with SEMA3A adenovirus. (**B**) Opposite patterns of expression of CDKs, P21 and P27 were observed in Sema3ASEMA3A-siRNA-transfected cells. Changes of protein expression were determined by scanning of the immunoreactive bands. For every cell type, the experimental group (Ad-SEMA3A or Si-SEMA3A) was compared to the matching control group (Ad-Con or Si-Scramble). Each data point represents the mean ± SD of three independent trials. *$P < 0.05$, **$P < 0.01$.

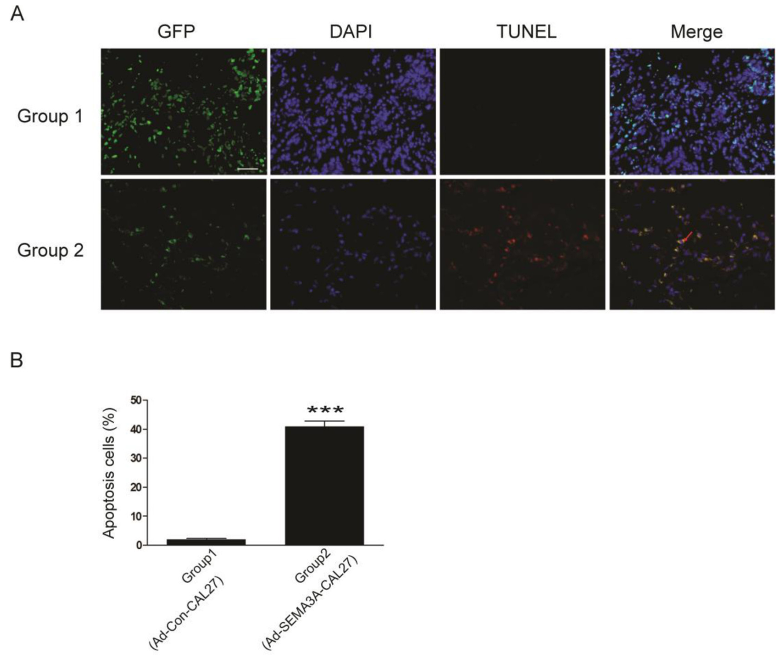

Supplementary Figure S4: SEMA3A over-expression induced apoptosis *in vivo*. (**A**) Mice with xenograft tumors (CAL27) were injected with Ad-Con adenovirus (group 1, n = 5) and Ad-SEMA3A adenovirus (group 2, n = 5) 2 times/week. After six weeks of injection, mice were sacrificed and tumors were dissected away, paraformaldehyde-fixed, paraffin-embedded and cut into sections. Apoptosis was assessed by TUNEL technique. Red arrow heads indicated apoptotic cells. Scale bar: 100 µm. (**B**) Apoptosis was quantified in the tumor lesions as percentage of TUNEL positive cells. The mice xenograft experiment was done for 3 times, with 5 mice in each group for each time. Results were expressed as mean ± SEM (group 1, n = 15; group2, n = 15). ***P < 0.001, t-test.

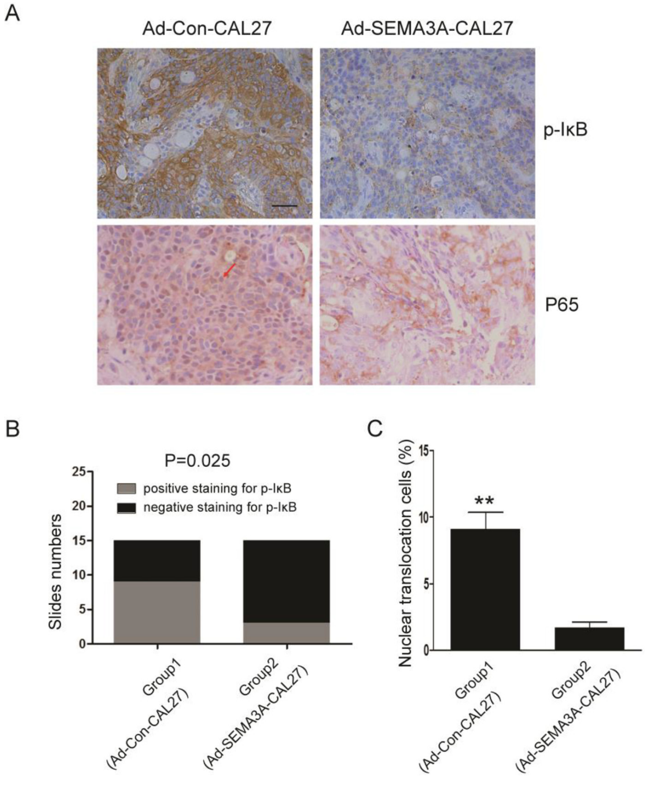

Supplementary Figure S5: SEMA3A over-expression induced decreased nuclear translocation of P65 and reduced p-IκB *in vivo*. (**A**) Freshly removed xenograft tumors were fixed and paraffin-embedded for histological analysis by IHC staining. Red arrow head indicated cells with nuclear translocation of P65. Scale bar: 100 µm. (**B**) p-IκB is highly expressed (9/15) in Group 1 tumors (Ad-Con-CAL27) and absent or reduced (3/15) in Group 2 tumors (Ad-SEMA3A-CAL27) (*P < 0.05, χ^2 test). (**C**) A total of 300 epithelial tumor cells were counted blindly by two independent observers from representative fields and the percentage of cells with nuclear translocation of P65 was recorded. All percentages were used to get the mean value per group. As shown, nuclear translocation of P65 of tissue cells in Group 1 was much higher than the cells in Group 2 (**P < 0.01, t - test).

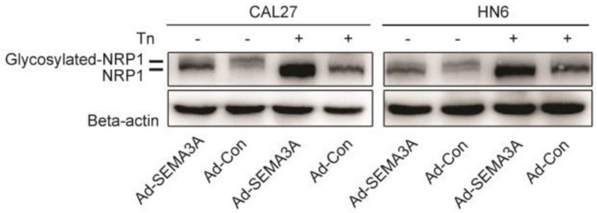

Supplementary Figure S6: SEMA3A over-expression induced decreased expression of the glycosylated form of NRP1. In CAL27 and HN6 cell lines, NRP1 was mainly expressed as a glycosylated form (upper faint bands, having a higher molecular weight due to glycosylation modification). After Ad-SEMA3A adenovirus infection, expression of the glycosylated form of NRP1 was significantly decreased, compared with Ad-Con cells (Tn-lanes). Cells after infection were then treated with tunicamycin (Tn, 5 μg/ml, sigma, USA), an antibiotic inhibitor of N-link glycosylation for 16 hours before harvesting. Blots of Tn+ lanes showed that upper bands of NRP1 were disappeared in Ad-Con cells while the lower bands of NRP1 in Ad-SEMA3A cells was increased. These results proved that the upper band was glycosylated NRP1.

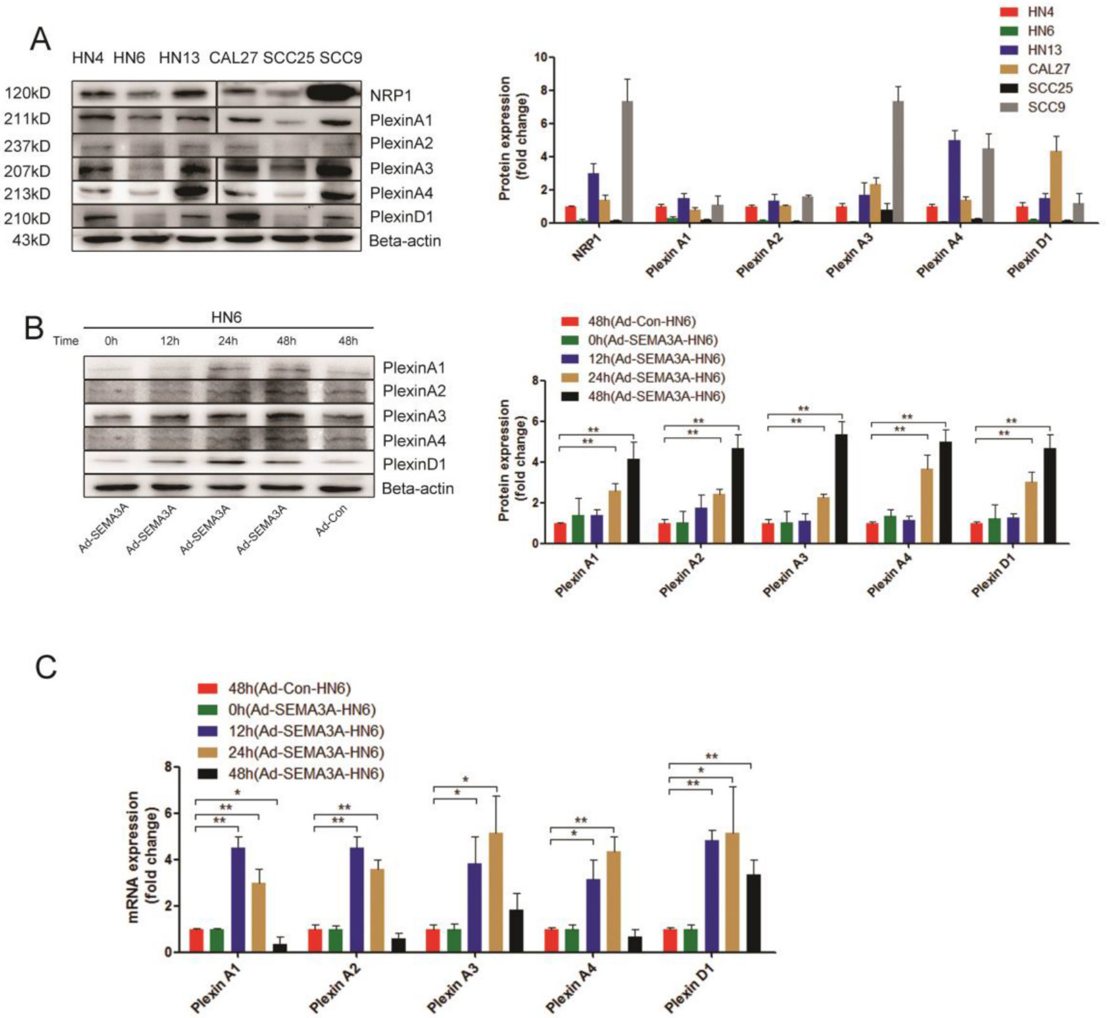

Supplementary Figure S7: Endogenous SEMA3A expression is partly identical to NRP1 and Plexins expressions in HNSCC cells and SEMA3A over-expression caused gradually increased Plexins. (**A**) The protein expression of SEMA3A has the same varying trend as that of NRP1 and Plexins (A1, 2, 3, 4, D1) as they all had a relatively high expression in HN4, HN13, SCC9, while had a low expression in HN6 and SCC25 cell lines. (**B**) Western blot analysis of protein with different harvest time (0 h, 12 h, 24 h and 48 h) after SEMA3A adenovirus transfection showed that Plexins were steadily increased as time extended. (**C**) The real-time PCR results revealed that expressions of Plexins (A1, 2, 3, 4, D1) mRNAs began to increase after SEMA3A adenovirus transfection until 36 h, after which Plexins (A1, 2, 3, 4, D1) mRNAs were decreased, which could be caused by the apoptosis of cells. Each data point represents the mean ± SD of three independent trials. *$P < 0.05$, **$P < 0.01$.
